# Eyes Front!

**Published:** 1920-01-03

**Authors:** 


### The Hospital

The Workers' Newspaper of Administrative Medicine and Institutional Life, Administration, National Insurance and Health.

No. 1752, Vol. LXVII. SATURDAY, JANUARY 3, 1920. PRICE TWOPENCE

### EYES FRONT!

The Old Year is slowly passing away as we write and the New Year, with its greater freedom, larger prospect for fulfilment, the awakenment of men and women of all classes to the great opportunities which present themselves for their development, and the new responsibilities which they must face and shoulder as worthy citizens of the reconstituted and extended Empire of which they form a part, invites their whole attention. Indeed, the experiences which the war has brought to millions of men and many women of our race should result in hundreds of thousands of them showing the fruits of a quickened intellect, and the possession of the faculty at present ascribed to wonderful people only, to whom each day opens a new life, never stale, but ever more stimulating and joyous than its predecessor.

We have before us a most remarkable instance in the person of H.R.H. the Prince of Wales, of a young man starting at twenty-five well-equipped to face the problems of life, to hold his own, and to exercise the widest and best of influences upon his fellows of all ages throughout the Empire. Such rare gifts as H.R.H. has shown himself to possess entail a heavy responsibility upon their possessor. We do, speaking from full conviction, and experience of the physician's point of view, express the most earnest hope that the grave dangers attaching to over-exertion, continuous excitement, and the discharge of unending public duties, should be recognised not only by the Prince, but by His Majesty the King, and all who are directly associated with him.

It should be seen that every safeguard and precaution are fully observed and strictly followed during the approaching busy, arduous months, or it may be years, which as the inheritor of a great position and responsibility must be entailed upon H.R.H. in the fulfilment of his duties as trustee for his people and their allies. The mortality tables of America, when studied carefully, reveal the incontrovertible fact that young men on the opening of their manhood, who are placed in positions of great responsibility entailing continuous application and an excessive amount of work day by day, are apt to shorten their lives by fully one-half of the allotted span of a normal man's life. The Prince of Wales' capacity for remarkable performance has been demonstrated to the hilt, and is admitted with gratitude by the whole Empire and all well-wishers of England. It would be indeed criminal if from any cause whatever his splendid health and manly development should be checked or stayed for the want of any adequate foresight, constant and devoted care, and the observance of laws which should secure and increase the powers and sound health of the ablest and best of men.

The nation and Empire are coming to recognise a home truth of splendid magnificence and value to our race. It is that through all the problems, trials, anxieties, dangers and threatenings of the worst war of the world, Buckingham Palace, despite its manifold and multitudinous duties of every conceivable kind, has never made one single mistake from beginning to end. Well may this nation sing with heart and soul "God Save the King," and bless all those closely associated with His Majesty who have rendered this glorious, nay unique, service, full of guarantees for the future, and redolent with hope and confidence too.

It is a remarkable feature of the moment that all classes of people, from the highest to the lowest, are displaying a keen interest in the adequate provision for the care and treatment of the sick, the wounded, and the permanently disabled throughout the nation and Empire. In the last few weeks The Hospital has dealt with several aspects of the questions which underlie the great problems here raised. It has obtained an official account from many hospital chairmen of the needs, progressive development, and present outlook of several of the largest and most important hospitals in Great Britain. These institutions were in danger of permanent injury by the silence of the Government in regard to its attitude towards the outcry by a relatively few people for the nationalisation of our hospitals. All uncertainty has been removed by Dr. Addison's, the Minister of Health, statements in reply to questions in the House of Commons on October 28, November 20, December 11 and 22, which will be found in another column.

Matters have developed rapidly so far as the voluntary hospitals are concerned, since the commencement of our campaign on December 8, and the progress made has been so great that we have good assurance that by the end of June next it is quite practicable to provide for the adequate financing of the voluntary hospitals, and to place them on the high road to permanent financial strength. The accomplishment of this glorious enterprise will afford an inspiring opportunity to every man and woman throughout Great Britain who takes an interest in public affairs, and is closely identified with the well-being, protection, and welfare of people in these islands. Heartening encouragement has come to us from important sources and, providing the people of all classes will extend to us their co-operation without delay, we have good grounds for hoping that the complete organisation of the nation in regard to its hospitals may be found practicable within the period named. There are still some chairmen who were written to before Christmas who have not yet responded in any way to the important letter addressed to them. We hope, however, that answers will be received to every letter we have issued, so as to reach us not later than January 14 at the latest. The course is clear. Thousands of people are already interested, and everything is tending in the direction of a great national movement, embracing the support of all classes of people, strong enough and representative enough to undertake and successfully accomplish this great enterprise. In such a case the voluntary hospital system will become adequate to supply all the needs of all classes of the population in regard to hospital care and treatment, their permanent restoration to health, and the people's continuous maintenance therein thenceforward.

